# The Prevalence of Parasites and Pathogens in Asian Honeybees *Apis cerana* in China

**DOI:** 10.1371/journal.pone.0047955

**Published:** 2012-11-07

**Authors:** Jilian Li, Haoran Qin, Jie Wu, Ben M. Sadd, Xiuhong Wang, Jay D. Evans, Wenjun Peng, Yanping Chen

**Affiliations:** 1 Key Laboratory of Pollinating Insect Biology of the Ministry of Agriculture, Institute of Apicultural Research, Chinese Academy of Agricultural Science, Beijing, China; 2 United States Department of Agriculture (USDA) – Agricultural Research Service (ARS) Bee Research Laboratory, Beltsville, Maryland, United States of America; 3 Experimental Ecology, Institute of Integrative Biology, ETH Zürich Universitätstrasse, Zürich, Switzerland; University of Utah, United States of America

## Abstract

Pathogens and parasites represent significant threats to the health and well-being of honeybee species that are key pollinators of agricultural crops and flowers worldwide. We conducted a nationwide survey to determine the occurrence and prevalence of pathogens and parasites in Asian honeybees, *Apis cerana*, in China. Our study provides evidence of infections of *A. cerana* by pathogenic *Deformed wing virus* (DWV), *Black queen cell virus* (BQCV), *Nosema ceranae*, and *C. bombi* species that have been linked to population declines of European honeybees, *A. mellifera*, and bumble bees. However, the prevalence of DWV, a virus that causes widespread infection in *A. mellifera*, was low, arguably a result of the greater ability of *A. cerana* to resist the ectoprasitic mite *Varroa destructor*, an efficient vector of DWV. Analyses of microbial communities from the *A. cerana* digestive tract showed that *Nosema* infection could have detrimental effects on the gut microbiota. Workers infected by *N. ceranae* tended to have lower bacterial quantities, with these differences being significant for the *Bifidobacterium* and *Pasteurellaceae* bacteria groups. The results of this nationwide screen show that parasites and pathogens that have caused serious problems in European honeybees can be found in native honeybee species kept in Asia. Environmental changes due to new agricultural practices and globalization may facilitate the spread of pathogens into new geographic areas. The foraging behavior of pollinators that are in close geographic proximity likely have played an important role in spreading of parasites and pathogens over to new hosts. Phylogenetic analyses provide insights into the movement and population structure of these parasites, suggesting a bidirectional flow of parasites among pollinators. The presence of these parasites and pathogens may have considerable implications for an observed population decline of Asian honeybees.

## Introduction

Honey bees are the most economically valuable pollinators in the world. In addition to producing hive products and pollinating crops such as clover and alfalfa that are used to feed cattle that produce meat and dairy, honey bees provide pollination service to one-third of crops that feed the world, It is estimated that the total economic value of pollination worldwide is €153 billion, representing 9.5% of the value of the world agricultural output for human food in 2005 [1]. However, increased colony losses have been reported from USA, Europe, and elsewhere over the recent decades [2]–[7]. The losses of honey bees especially the Colony Collapse Disorder (CCD), a serious malady that resulted in a loss of 50–90% of colonies in beekeeping operations across the U.S. in 2007 [8]–[9] pose a serious threat to the agricultural and natural ecosystems that rely on honey bees for pollination. A combination of multiple stressors including pesticide exposure, inadequate nutrition, and microbial diseases has been suggested to set off a cascade of harmful effects and contribute to increased colony losses [10]–[12]. While the exact cause of colony losses remains unidentified, a growing body of evidence has indicated that parasites and pathogens are key culprits implicated in massive disappearance/death and population declines of honeybees [8], [10]–[15].

The asiatic honeybee (*Apis cerana*) has a long apicultural history. While it is still found in the wild, *A. cerana* is one of the few bee species that can be domesticated and used in apiculture. Compared to European honey bees *A. mellifera*, *A. cerana* has a strong ability to work in cold temperatures as low as −0.1°C that could be lethal to *A. mellifera*. In addition, *A. cerana* possesses behavioral and physiological mechanisms to defend against the ectoparasitic mite, *Varroa destructor* [16], a species that has had a catastrophic effect on the population of *A. mellifera*. Further, *A. cerana* can fly several kilometers on foraging trips allowing this species to utilize sporadic nectar sources [17]. With these advantages, *A. cerana* is a vitally important pollinator of crops and flora in mountainous areas and plays a critical role in enhancing biodiversity conservation [18]–[19]. In recent years, population sizes of *A. cerana* have rapidly decreased because of extensive use of pesticides, changes of plant biodiversity, loss of habitats, competition with non-native species, and inter-species transfer of pathogens and parasites [17], [19]–[20]. Consequently, there is an acute need for investigating the causes of the bee losses and for finding novel solutions for bee health problems.

China has a long history of bee culture and is one of the largest beekeeping countries in the world and leads the world in the number of bee colonies and honey production. Before the introduction of the exotic honeybee, *A. mellifera ligustica*, in 1893, *A. cerana* was the main pollinator of crops and natural flora. *A. mellifera* and *A. cerana* are now present throughout China and share many common foraging habitats over a huge geographical area. While both laboratory and field studies have been undertaken to investigate the cause or causes of colony collapse of *A. mellifera* in many parts of world, few studies were conducted to explore reasons for *A. cerana* declines [19]. Due to the importance of parasites and pathogens in relation to the health of honey bee colonies, we used molecular genetic analyses to carry out the first nationwide survey of honey bee pathogens and parasites linked to *A. mellifera* colony collapse in Chinese *A. cerana*. In addition, we measured the distribution and prevalence in *A. cerana* of the trypanosome relative of *Crithidia bombi*, a gut parasite of bumblebees that has been implicated in declining bumble bee populations [20]–[21]. In natural systems, most disease agents are capable of infecting multiple host species and pathogenic microorganisms that utilize multiple hosts are more often implicated in emergent diseases of animals [22]–[24]. Previous studies showed that parasites and pathogens attacking honeybees could be transmitted between different host species. For instance, *Melissococcus plutonius*, a bacterium that infects *A. mellifera* larvae leading to European foulbrood disease, caused serious damage to colonies of *A. cerana* in China from 1972 to 1976 [17]. Further, viruses that are frequent in *A. mellifera* have been identified in *A. cerana* [25]–[26] and different species of bumble bees [27]–[29], as well as in eleven non-apis hymenopteran species [30]. On the other hand, *V. destructor* was transmitted from its original host *A. cerana* to *A. mellifera* in the middle of the 20th century [31] and has caused catastrophic damage to *A. mellifera* since then. More recently, a microsporidian gut parasite *Nosema ceranae* that was first discovered in *A. cerana* has emerged as a potentially virulent pathogen of *A. mellifera*. *N. ceranae* has been associated with colony collapse of *A. mellifera* and recently extended its host range to bumble bees [32]–[33]. Therefore, a survey to investigate the prevalence of disease-causing pathogens/parasites in populations of *Apis cerana* would allow us to gain critical insights into the pathogen/parasite-host interactions and to determine the roles of pathogens and parasites on observed population decline of *Apis cerana* in China. Not all microorganisms within a host will play a parasitic role. Indeed, microbes within organisms, and particularly those within the gut, have been receiving increasing attention because of the beneficial functions they can play for their hosts. These microbes can help to digest food [34]–[36], synthesize nutrients [37]–[38], degrade ingested toxins [39], and provide defenses against parasites [40]–[41]. Some studies on bacteria associated with honeybees and bumblebees have been reported, with studies showing a relatively low diversity of conserved gut microbes and consisting of eight phylogentically close species or phylotypes that share ≥97% sequence identify in 16S rRNA sequences [42]–[49]. Furthermore, at least in bumblebees, it has been demonstrated that this microbiota is important in host resistance to a gut-infecting parasite [50]. Due to the potentially important link between gut parasites and gut symbionts, we compare bacterial loads of focal bacteria within the honeybee microbiota between workers infected or not infected with *N. ceranae*. Our data provide a snapshot of the prevalence of parasites in Chinese *A. cerana*, and show the potential for the spread of parasites to new locations and other host species in China and elsewhere.

## Materials and Methods

### 1. Bee sampling

**Figure 1:**
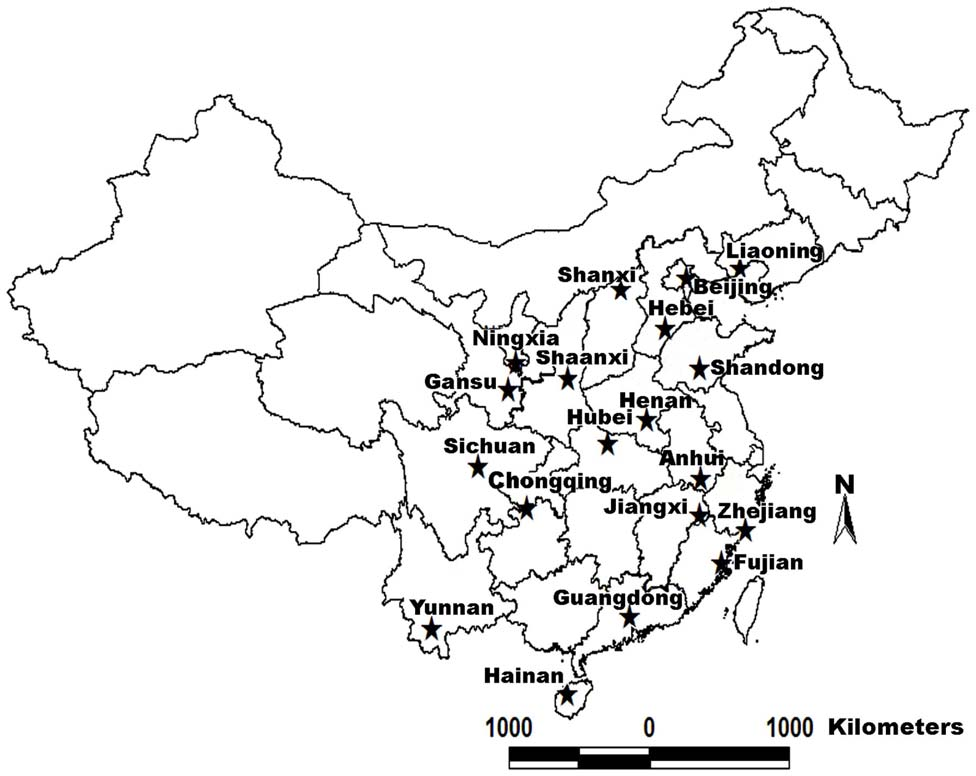
Sampling locations in China. See Table 2 for collection details of location.

*A. cerana* worker samples were collected in 19 provinces in China (Figure 1) between November 2010 and January 2011. Three bee colonies were analyzed per province, and 40 worker bees screened for parasites and pathogens from each colony. A total of 1140 worker samples were collected from both managed and wild colonies. Half of the samples were used for isolation of DNA and examination of gut parasites *N. ceranae* and *C. bombi*. The remaining samples were used for RNA isolation and viral RNA testing. All samples were alive when collected and shipped to the laboratory in small cages containing sugar powder prior to storage at −80°C until processing.

### 2. RNA Isolation and RT-PCR Detection

Total RNA was isolated from individual honeybees using the TRIzol reagent (Invitrogen) according to the manufacturer's instructions. All RNA samples were tested for the presence of seven viruses commonly found in *A. mellifera* including *Acute bee paralysis virus* (ABPV), *Black queen cell virus* (BQCV), *Chronic bee paralysis virus* (CBPV), *Deformed wing virus* (DWV), *Kashmir bee virus* (KBV), *Israeli acute bee paralysis virus* (IAPV), and *Sacbrood virus* (SBV). Amplification of specific viral RNAs was carried out using the Access RT-PCR kit (Promega, Madison, WI) according to the manufacturer's instructions. Previously published primers for virus detection in *A. mellifera* were used and shown in Table 1. The reaction mixture contained: 1× AMV/Tfl reaction buffer, 0.2 mM each deoxynucleotide triphosphate (dNTP), 1 µM of sense primer, 1 µM of antisense primer, 2 mM MgSO_4_, 0.1 unit AMV reverse transcriptase, 0.1 unit T*fl* DNA polymerase and 500 ng total RNA in a total volume of 25 µl. Amplification was undertaken with the following thermal cycling profiles: one cycle at 48°C for 45 min for reverse transcription; one cycle of 95°C for 2 min; 40 cycles at 95°C for 30 s, 55°C for 1 min, and 68°C for 2 min; and one cycle of 68°C for 7 min. Negative (water) and positive control (recombinant plasmid DNA with virus insert into pCR 2.1 vector) were included in each run of the RT-PCR reaction. PCR products were electrophoresed in 1% agarose gel containing GoldView(GV) and visualized under UV light. PCR products were purified using the Agarose Gel DNA Purification kit Ver.2.0 (Takara, Japan) and sequenced to ensure their specificity.

**Table 1:** Primers used for pathogen/parasite and microbiota detection and quantification.

Organism	Species	Primers	Size (bp)	References
Virus	Acute Paralysis Bee Virus	ABPV-F:(5-ttatgtgtccagagactgtatcca-3)ABPV-R: (5-gctcctattgctcggtttttcggt-3)	900	[66]
Virus	Chronic Bee Paralysis Virus	CBPV-F:(5-agttgtcatggttaacaggatacgag-3)CBPV-R: (5-tctaatcttagcacgaaagccgag-3)	455	[67]
Virus	Black Queen Cell Virus	BQCV-F:(5-tggtcagctcccactaccttaaac-3)BQCV-R: (5-gcaacaagaagaaacgtaaaccac-3)	700	[66]
Virus	Deformed Wing Virus	DWV-F: (5-atcagcgcttagtggaggaa-3)DWV-R: (5-tcgacaattttcggacatca-3)	702	[68]
Virus	Kashmir Bee Virus	KBV-F: (5-gatgaacgtcgacctattga-3)KBV-R: (5-tgtgggttggctatgagtca-3)	415	[69]
Virus	Sacbrood	SBV-F: (5-gctgaggtaggatctttgcgt-3)SBV-R: (5-tcatcatcttcaccatccga-3)	824	[70]
*Microsporidium*	*N.apis*	N.apis-F:(5-ccattgccggataagagagt-3)N.apis-R:(5-ccaccaaaaactcccaagag-3)	269	
*Microsporidium*	*N.ceranae*	N.ceranae-F (5-gacaacaaggaagacctggaagtg-3)N.ceranae-R: (5-tgtgaataagagggtgatcctgttgag-3)	780	[60]
Trypanosome	Crithidia	Crith-F(5-ggaaaccacggaatcacatagacc-3)Crith-R(5-aggaagccaagtcatccatcgc-3)	500	
Bacterium	Bact774	Bac-774F(5-gtagtccacgctgtaaacgatg-3)Bac-1391R(5-gacgggcggtgtgtrca-3)	900	[10]
Bacterium	Neisseriaceae	Neiss-F(5-aagcggtggatgatgtgg-3)Neiss-R(5-tgatggcaactaatgacaagg-3)	197	
Bacterium	Pasteurellaceae	Past-F(5-ttgttgccagcgattagg-3)Past-R(5-attctgattcacgattactagc-3)	243	
Bacterium	Bifidobacterium	Bifi-F(5-caagcgagagtgagtgtacc-3)Bifi-R(5-gccgatccaccgttaagc-3)	165	
Bacterium	Lactobacillus	Lact-F(5-taacgcattaagcactcc-3)Lact-R(5-gctggcaactaataataagg-3)	270	
*Apis cerana*	*Apis*-β-actin	Actin-F(5-aggaatggaagcttgcggta-3)Actin-R(5-aattttcatggtggatggtgc-3)	181	

### 3. DNA Isolation and PCR Detection

The abdomen of 570 individual bees was removed with scissors and individually homogenized in 100 µl of Krebs Ringer solution with a sterile pestle individually. Total genomic DNA was extracted from 50 µl of the homogenate of the single bee abdomen using a DNA purification kit (Wizard® SV 96 Genomic DNA Purification System (Promega). DNA samples were stored at −20°C prior to molecular screening for parasites. Primers used for detection of *N. ceranae*, *N. apis* and *C. bombi* are listed in Table 1. PCRs were performed using a Mastercycler 5333 (Eppendorf) in 25 µl volumes containing 500 ng of template DNA, 2.5 µl of 10×PCR buffer, 2.0 µl of dNTPs (200 µM), 0.25 µl of *Ex Taq* polymerase (TaKaRa, Co.Ltd.), and 1 µl of each forward and reverse primers (10 µM), plus 13.65 µl of water. Amplification was undertaken with the following thermal cycling profiles: initial DNA denaturation step of 4 min at 94°C followed by 40 cycles of 30 s at 94°C, 30 s at 56°C, and 60 s at 72°C, and terminated with a final extension step of 72°C for 10 min. For each run of the PCR reaction, negative (water) and positive (previously identified positive sample) controls were run along with DNA extracts of samples. PCR products were electrophoresed in a 1.2% agarose gels containing 0.5 ug/ml GoldView(GV) and visualized under UV light. Some of the PCR-amplified bands were purified and sequenced to verify their identities. Obtained DNA sequences were used for constructing phylogenetic trees.

### 4. Bacteria detection and real time quantitative PCR (qPCR)

To assess the abundance of symbiotic bacteria in *A. cerana*, DNA samples from worker bees of 19 provinces were mixed together and PCR-amplified with universal eubacterial primers 774F and 1391R (Table 1) for the detection of gut bacteria. The amplification program was as follows: initial DNA denaturing step of 4 min at 94°C followed by 32 cycles of 30 s at 94°C, 30 s at 56°C, and 60 s at 72°C, and terminated with a final extension step of 72°C for 10 min. PCR products were purified using the Agarose Gel DNA Purification kit Ver.2.0 (Takara, Japan) and ligated into the pMD19-T vector (Takara, Japan). The recombinant plasmid DNA was then transformed into bacteria competent cells and the competent cells from transformation were plated onto LB plates with ampicillin and tetracycline to select for transformants and X-gal (5-bromo-4-chloro-3-indoly-b-D-galactopyranoside) and IPTG (isopropyl-b-D-thiogalactopyranoside) for blue/white colony selection. A total of 180 clones were picked and sequenced using M13-reverse and M13-forward universal primers in an automated fluorescence sequencing system ABI (Perkin–Elmer) with a Big-dye terminator v3.0 Cycle Sequencing Ready Reaction for an ABI Prism 310 Genetic Analyzer (Applied Biosystems). Sequences obtained were BLAST analyzed and matched with valid reference sequences in the NCBI (National Center for Biotechnology Information) to determine the bacterial species types. The specific bacteria primers were designed by using Beacon designer 7.5 based on sequencing of bacterial taxonomic groups obtained from the clone library. The specific bacteria primers and internal control primer β-actin are shown in Table 1. SYBR Green real-time quantitative PCR (qPCR) was used to compare bacterial loads between bees infected with *N. ceranae* and those that did not have this parasite. Samples that were infected with *N. ceranae* and not infected with *N. ceranae* from different provinces were separated into two groups and examined for presence and abundance of bacteria. qPCR was performed using an Mx3005P real-time PCR system operated by MxPro qPCR software (Stratagene, La Jolla, CA). To ensure the effectiveness of the nucleic acid extraction and amplification, real-time qPCR targeting the house keeping gene β-actin (Table 1) was used for each sample. The thermal profile parameters consisted of one cycle at 95°C for 3 min followed by 35 cycles of 95°C for 30 sec, 55°C for 1 min, and 72°C for 30 sec. Negative control (no template) was included in each run of the reaction. The positive control was purposely not included in the reaction in order to avoid any potential chances of contamination. After amplification, a melting curve analysis was performed to determine the specificity of the PCR products. The PCR products were incubated for 1 min at 95°C, ramping down to 55°C at a rate of 0.2°C/sec. The dissociation curve was constructed using 81 complete cycles of incubation where the temperature was increased by 0.5°C/cycle, beginning at 55°C and ending at 95°C. The expected size of the PCR product was confirmed by 1.5% agarose gel electrophoresis and subsequent visualization with ethidium bromide. Individual PCR bands were cut off and purified using Wizard PCR Prep DNA Purification System (Promega, Madison, WI) and sequenced to confirm the specificity of the qPCR assay. The nucleotide sequences of PCR products were determined and compared with sequences published at GenBank, National Center for Biotechnology Information, NIH. The level of each bacterial species group and β-actin was quantified based on the value of the cycle threshold (Ct; the number of cycles needed to generate a fluorescent signal above a predefined threshold). qPCR was replicated three times for each sample to address experimental error. The output of qPCR assays was interpreted by using the comparative Ct method (ΔΔCt Method). The average Ct value (ΔCt) of each bacterial species was normalized using the Ct value corresponding to the β-actin following the formula: ΔCt = (Average Ct_target_)−(Average Ct_β-actin_). *N. ceranae* infected bees had relatively lower level of titers for each bacterial types compared to non-infected workers and therefore were chosen as a calibrator. The concentration of each bacterial group in non-infected bees was compared with calibrator and expressed as n-fold change. The ΔCt value of each bacterial type in non-infected workers was subtracted by that of workers infected by *N. ceranae* to yield ΔΔCt value. The fold difference in quantities of each bacterial type between non-infected workers and *N. ceranae* infected workers was calculated using the formula 2^−ΔΔCt^.

### 5. Statistical analyses

Statistical analysis was carried out in SPSS 17.0 (SPSS Inc.,Chicago, IL, US). The infection prevalence of parasites was compared among different provinces using a Chi-square test. The correlations between *N. ceranae* and *C. bombi* was analyzed as infection or non-infection of individual bees (n = 570). BQCV and DWV prevalence was analysed per province (n = 19) using a Kendall tau rank correlation. The significant difference of each bacterial load between *N. ceranae* infected and non-infected workers with SYBR-Green used for detection was analysed using a one sample t-test.

### 6. Phylogenetic analysis

The sequence data were aligned by ClustalX using default settings [51] and visually checked using BioEdit [52], followed by a BLAST database search to test sequence similarities [53]. Phylogenetic analysis was conducted in MEGA5 using ClustalW and a neighbor-joining algorithm. Phylogenies were assessed by bootstrap replication (N = 500 replicates). Numbers at nodes correspond to bootstrap percentages with values of greater than 50 percent being regarded as providing evidence for the phylogenetic grouping.

## Results

### 1. Geographic distribution of major crops and flora across the China

China has abundant resources of honey plants, so far 9800 kinds of honey plants are available, belong to 110 families and 394 genera, including crops, fruit trees, vegetables, melons, pasture, forest plants, medical plants, spice crops. Among these plants, about 30 kinds of honey plants are used to generate most number of commercial honey [54]. In this paper, honeybee samples were collected from areas with the following major floral plants: Chinese milk vetch (Hebei, Anhui, Jiangxi, Zhejing), Locust (Beijing, Henan, Shandong), Linden (Liaoning), Chaste tree (Beijing, Henan), Citrus (Zhejing, Guangdong, Fujian), Lychee (Guangdong, Hainan, Fujian), Longan (Guangdong, Hainan, Fujian), Rape (Hebei, Shanxi, Sichuan, Gansu, Ningxia), Russian olive(Shaanxi), Buckwheat (Gansu, Ningxia), Elsholtzia rugulosa (Yunnan), Coconut tree (Hainan).

### 2. Virus frequencies in *A. cerana* colonies

Prevalence data for seven bee viruses screened in 570 worker bees from 19 provinces are shown in Table 2. Of these viruses, only BQCV and DWV were detected in worker bees. BQCV was found in six of 19 provinces. The highest infection rate of 98% was found in Gansu and the lowest infection rate of 12% was found in Henan province, with a significant difference between provinces in prevalence of the virus (x^2^ = 433.622, *P* = 0.001; Table 2). DWV was detected only in three provinces, with the highest and lowest infection rates of 92 and 33%, respectively, found in Fujian and Sichuan provinces (Table 2). Despite the low occurrences across provinces, there was a significant correlation between the prevalence of BQCV and DWV across the different provinces (Kendall's tau rank: r = 0.508, n = 19, p = 0.018).

**Table 2:** Synopsis of collection details and prevalence of parasites in *A. cerana* colonies from 19 provinces in China.

Province	Latitude N	Longitude E	Prevalence of parasites (95%CI)							Prevalence of viruses (95%CI)						
			N	*N ceranae*	x^2^	*P*	*C. bombispp.*	x^2^	*P*	N	BQCV	x^2^	*P*	DWV	x^2^	*P*
**N. CHINA**																
Beijing	40.13	117.1	30	0.23(0.09–0.38)	4.55	0.03	0.23(0.09–0.38)	2.71	0.10	30	0	6.63	0.01	0	1.90	0.17
Hebei	37.77	114.52	30	0.36(0.12–0.41)	0.49	0.48	0.33(0.05–0.55)	0.10	0.76	30	0	6.63	0.01	0	1.90	0.17
Shanxi	39.54	112.93	30	0.33(0.19–0.48)	1.12	0.29	0.14(0.02–0.31)	2.71	0.01	30	0	6.63	0.01	0	1.90	0.17
Henan	35.2	113.8	30	0.57(0.16–1.79)	1.15	0.28	0	10.57	0.001	30	0.12(0.01–0.28)	0.62	0.43	0.51(0.25–0.75)	44.96	0.001
Shandong	35.71	117.92	30	0.24(0.09–0.38)	4.55	0.03	0	10.57	0.001	30	0	6.63	0.01	0	1.90	0.17
Gansu	34.62	105.7	30	0.27(0.02–0.55)	3.15	0.08	0	10.57	0.001	30	0.98 (0.82–1.11)	83.29	0.001	0	1.90	0.17
Ningxia	36.01	106.2	30	0.60(0.20–0.80)	2.14	0.15	0	10.57	0.001	30	0	6.63	0.01	0	1.90	0.17
Shaanxi	38.2	109.7	30	0.18(0.04–0.44)	8.14	0.004	0	10.57	0.001	30	0	6.63	0.01	0	1.90	0.17
Liaoning	40.62	120.52	30	0.82(0.69–0.98)	15.39	0.001	0.22(0.05–0.52)	0.21	0.65	30	0	6.63	0.01	0	1.90	0.17
**S. CHINA**																
Anhui	30.2	118.01	30	0.23(0.03–0.80)	4.55	0.03	0.63(0.49–0.78)	14.27	0.001	30	0	6.63	0.01	0	1.90	0.17
Jiangxi	28.4	117.9	30	0.17(0.11–0.65)	8.14	0.004	0.75(0.59–0.88)	27.99	0.001	30	0.91(0.65–1.15)	69.56	0.001	0	1.90	0.17
Zhejiang	30.2	120.01	30	0.92(0.65–1.22)	24.90	0.001	0.12(0.01–0.35)	2.71	0.01	30	0	6.63	0.01	0	1.90	0.17
Guangdong	23.33	113.5	30	0.73(0.16–1.31)	8.15	0.004	0.13(0.01–0.28)	2.70	0.01	30	0	6.63	0.01	0	1.90	0.17
Hainan	19.5	109.5	30	0.35(0.04–1.17)	0.49	0.49	0.32(0.05–0.55)	0.11	0.77	30	0	6.63	0.01	0	1.90	0.17
Hubei	32.01	112.1	30	0.15(0.04–0.70)	10.32	0.001	0.97(0.82–1.11)	56.69	0.001	30	0.35 (0.22–0.51)	3.20	0.07	0	1.90	0.17
Fujian	26.01	119.3	30	0.83(0.65–1.80)	15.39	0.001	0.73(0.59–0.88)	24.15	0.001	30	0.73(0.59–0.88)	40.27	0.001	0.92(0.65–1.15)	170.21	0.001
Sichuan	30.6	104.01	30	0.13(0.02–0.51)	10.32	0.001	0	10.57	0.001	30	0.92(0.65–1.22)	69.55	0.001	0.33(0.15–0.65)	15.98	0.001
Yunnan	25.52	102.41	30	0.54(0.09–1.16)	0.51	0.48	0.76(0.62–0.91)	27.99	0.001	30	0	6.63	0.01	0	1.90	0.17
Chongqing	28.77	106.9	30	0.98(0.82–1.11)	28.57	0.001	0.36(0.01–0.75)	0.49	0.49	30	0	6.63	0.01	0	1.90	0.17
**TOTAL**			**570**	**0.45(0.37–0.55)**	**173.02**	**0.001**	**0.29(0.20–0.38)**	**263.89**	**0.001**	**570**	**0.21(0.12–0.31)**	**433.62**	**0.001**	**0.09(0.03–0.16)**	**379.89**	**0.001**

### 3. Microsporidian and trypanosome parasite frequencies in *A. cerana* colonies

The data for *N. apis*, *N. ceranae*, and trypanosome prevalence is summarized in Table 2. *N. ceranae* was detected in *A. cerana* workers from 19 provinces. In general, Southern part of provinces had a relatively higher infection rate than Northern part of provinces. The average infection rate of *N. ceranae* in Southern provinces was 50.3% while the average infection rate of *N. ceranae* in Northern provinces was 40%. Among provinces with detectable *N. ceranae*, the highest prevalence for *N. ceranae* was found in Chongqing, with infection rate of 98%. The lowest prevalence of *N. ceranae* was found in Sichuan with infection rate of 13%. *N. apis* was not detected in any samples examined (Table 2). *C. bombi* was detected in *A. cerana* workers from 13 provinces. The highest infection of *C. bombi* was found in Hubei province with infection of 97%, and the lowest infection was in Zhejiang with infection rate of 12% (Table 2). Both *N. ceranae* (x^2^ = 173.02, *P* = 0.001) and *C. bombi*. (x^2^ = 263.89, *P* = 0.001) were found at differing proportions across provinces. Interestingly, there was a negative correlation between the incidence of *N. ceranae* and *C. bombi* across provinces (Kendall's tau rank: r = 0.09, n = 570, p = 0.004).

### 4. Phylogenetic relationship

**Figure 2:**
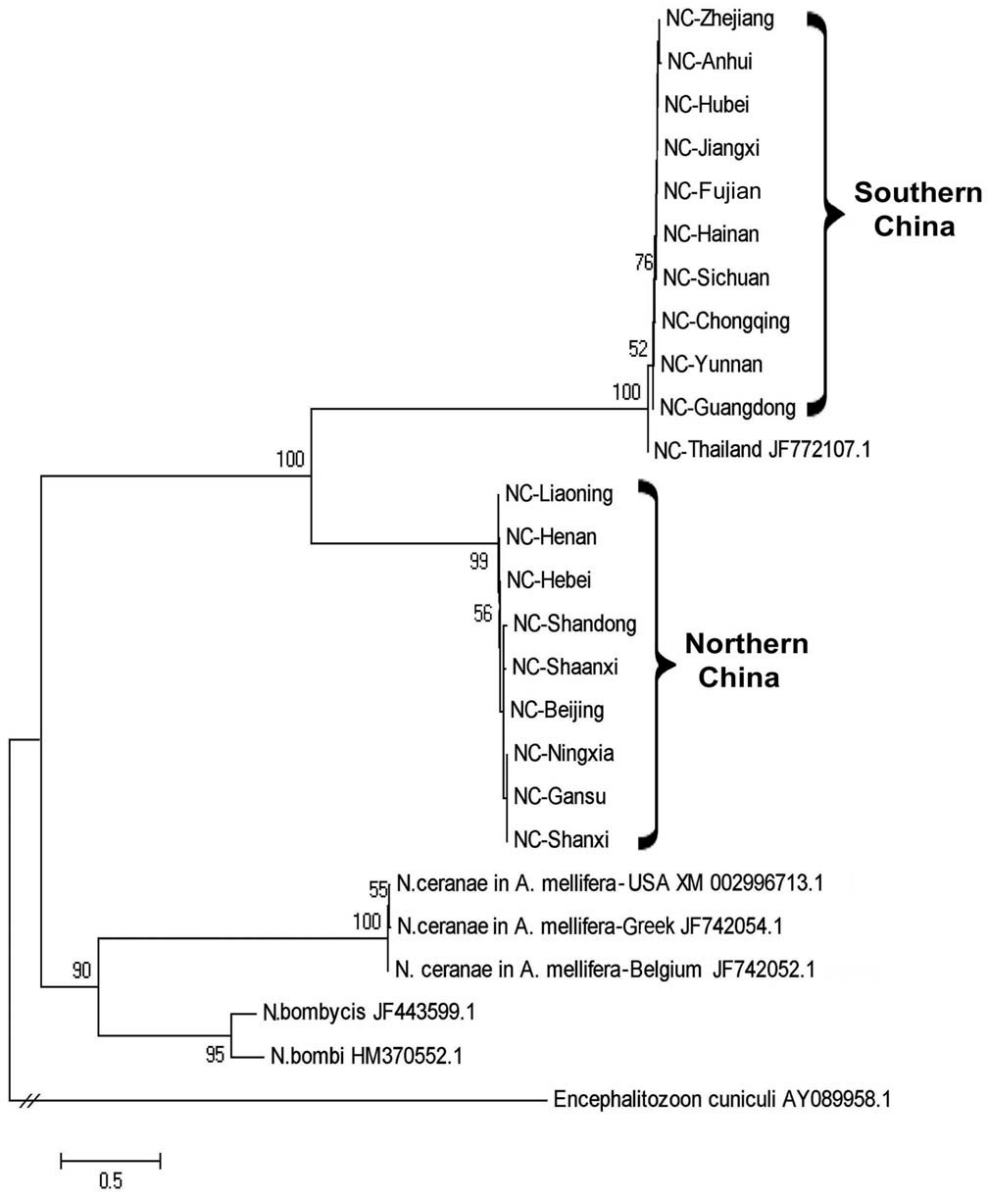
A phylogenetic tree showing the relationship of *Nosema ceranae* isolates. The partial sequences of 16S ribosomal RNA of *N. ceranae* from *A. cerana* collected in different geographic locations of China and from *A. mellifera* retrieved from GenBank were aligned using ClustalW. The tree was built using the Neighbor-Joining method. The sequence of *Encephalitozoon cuniculi* was used as an outgroup to root the tree. Numbers at each node represent bootstrap values as percentages of 500 and only bootstrap values greater than 50% are shown.

**Figure 3:**
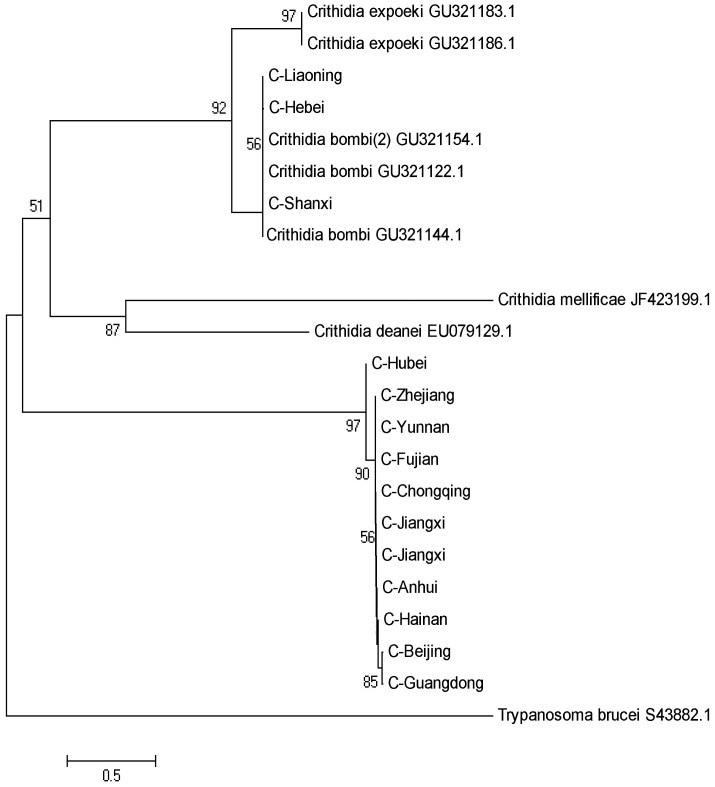
A phylogenetic tree showing the relationship of *C. bombi* isolates. The partial sequences of 18 S ribosomal RNA of *C. bombi* from *Apis cerana* collected from different geographic locations of China and from bumble bee species retrieved from GenBank were aligned using ClustalW. The tree was built using the Neighbor-Joining method. The sequence of *Trypanosoma brucei* was used as an outgroup to root the tree. Numbers at each node represent bootstrap values as percentages of 500 and only bootstrap values greater than 50% are shown.

**Figure 4:**
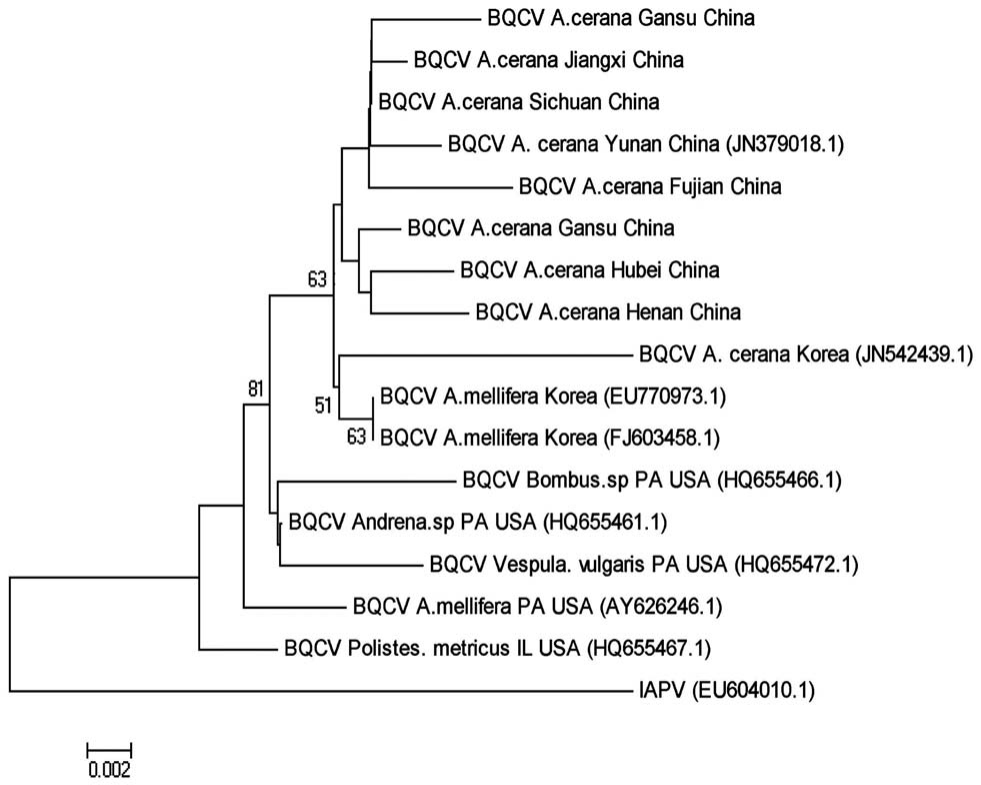
A phylogenetic tree showing the relationship of BQCV isolates. The partial sequences of 3′ UTR of BQCV from *A. cerana* collected from different geographic locations of China and from *Apis mellifera* retrieved from GenBank were aligned using ClustalW. The sequence of IAPV was used as an outgroup to root the tree. Numbers at each node represent bootstrap values as percentages of 500 and only bootstrap values greater than 50% are shown.

**Figure 5:**
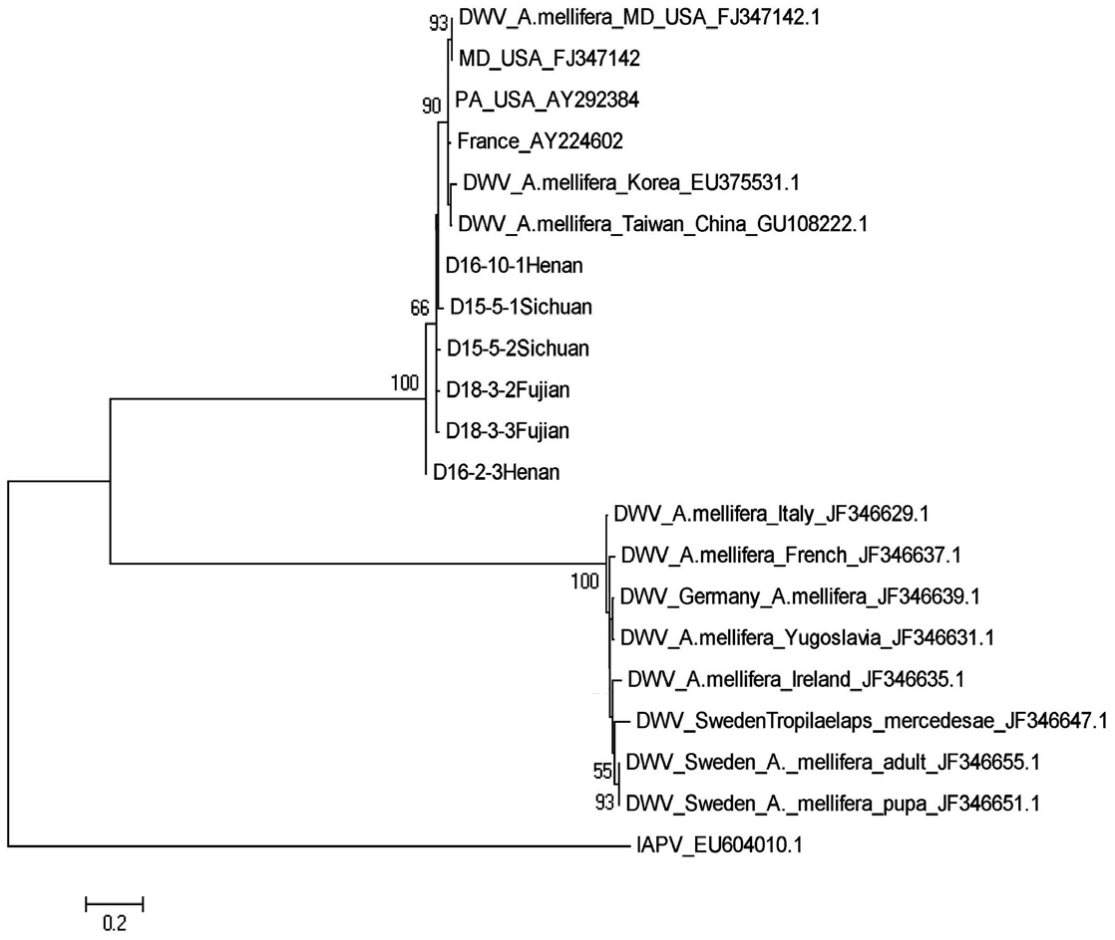
A phylogenetic tree showing the relationship of DWV isolates. The partial sequences of RNA dependent RNA polymerase (RdRp) of DWV from *A. cerana* collected from different geographic locations of China and from *Apis mellifera* retrieved from GenBank were aligned using ClustalW. The sequence of IAPV was used as an outgroup to root the tree. Numbers at each node represent bootstrap values as percentages of 500 and only bootstrap values greater than 50% are shown.

Phylogenetic trees were constructed to assess relationship between the samples of *N. ceranae* and *C. bombi* from different geographic locations. The *N. ceranae* isolates from the north and south regions of China separate into two distinct lineages, both of which are different from the *N. ceranae* strain now circulating in *A. mellifera*. The *N. ceranae* isolate from Thailand falls into the same clade with the isolates from south region of China (Figure 2). *C. bombi* isolates from *A. cerana* colonies were also separated into two clades with sequences of *C. bombi* from *A. cerana that* were collected from the northern provinces of China, Hebei, Liaoning, and Shanxi nearly identical to *C. bombi* isolated from bumble bees and isolates from *A. cerana* from the southern region of China clustering together (Figure 3). Phylogenetic trees based on the partial sequences of the 3′UTR of BQCV and of RNA-dependent RNA polymerase (RdRp) of DWV were constructed to illustrate the genetic relationship between viral isolates from *A. cerana* collected in different parts of China and existing GenBank accessions from *A. mellifera*. BQCV isolates infecting *A. mellifera* constituted the early lineages of the *Apis*-based clade and BQCV isolates from *A. cerana* in China clustered together. One sequenced isolate from *A. cerana* in Korea was distinct from the others (Figure 4). Sequence divergence among BQCV isolates did not correspond with the locations of provinces of China (Figure 4). A phylogenetic tree of DWV isolates demonstrated that DWV isolated from *A. cerana* is homogeneous and nearly identical to one of two distinct DWV lineages found in *A. mellifera* (Figure 5).

### 5. Bacteria detection and quantification in *A. cerana* colonies

**Figure 6:**
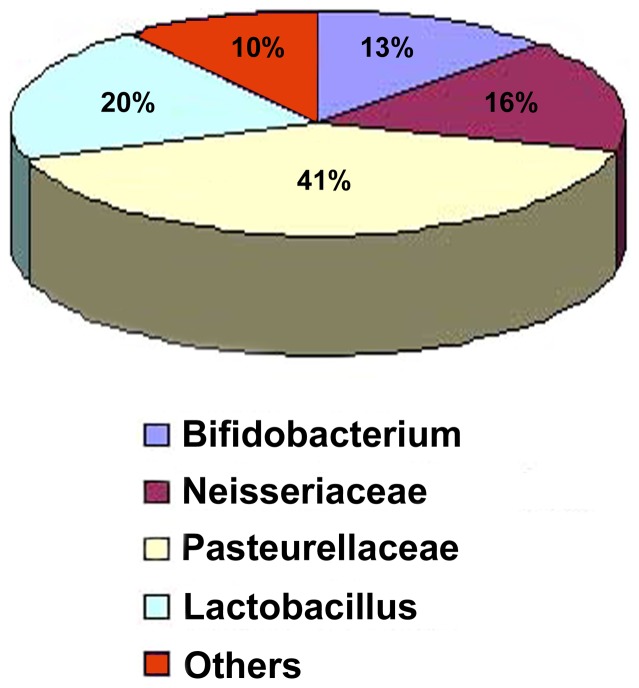
Pie chart showing the percentage of Bifidobacterium, Neisseriaceae, Pasteurellaceae and Lactobacillus and uncommon bacteria types identified in worker bees.

DNA PCR detection in the workers infected and non-infected by *N. ceranae* identified four most commonly identified bacterial species groups including *Bifidobacterium*, *Neisseriaceae*, *Pasteurellaceae* and *Lactobacillus*. Of bacteria types identified from clone library screening, 41% were *Pasteurellaceae*, 20% were *Lactobacillus*, 16% were *Neisseriaceae*, 13% were *Bifidobacterium and* 10% were mixture of uncommon bacteria types (Figure 6).

**Figure 7:**
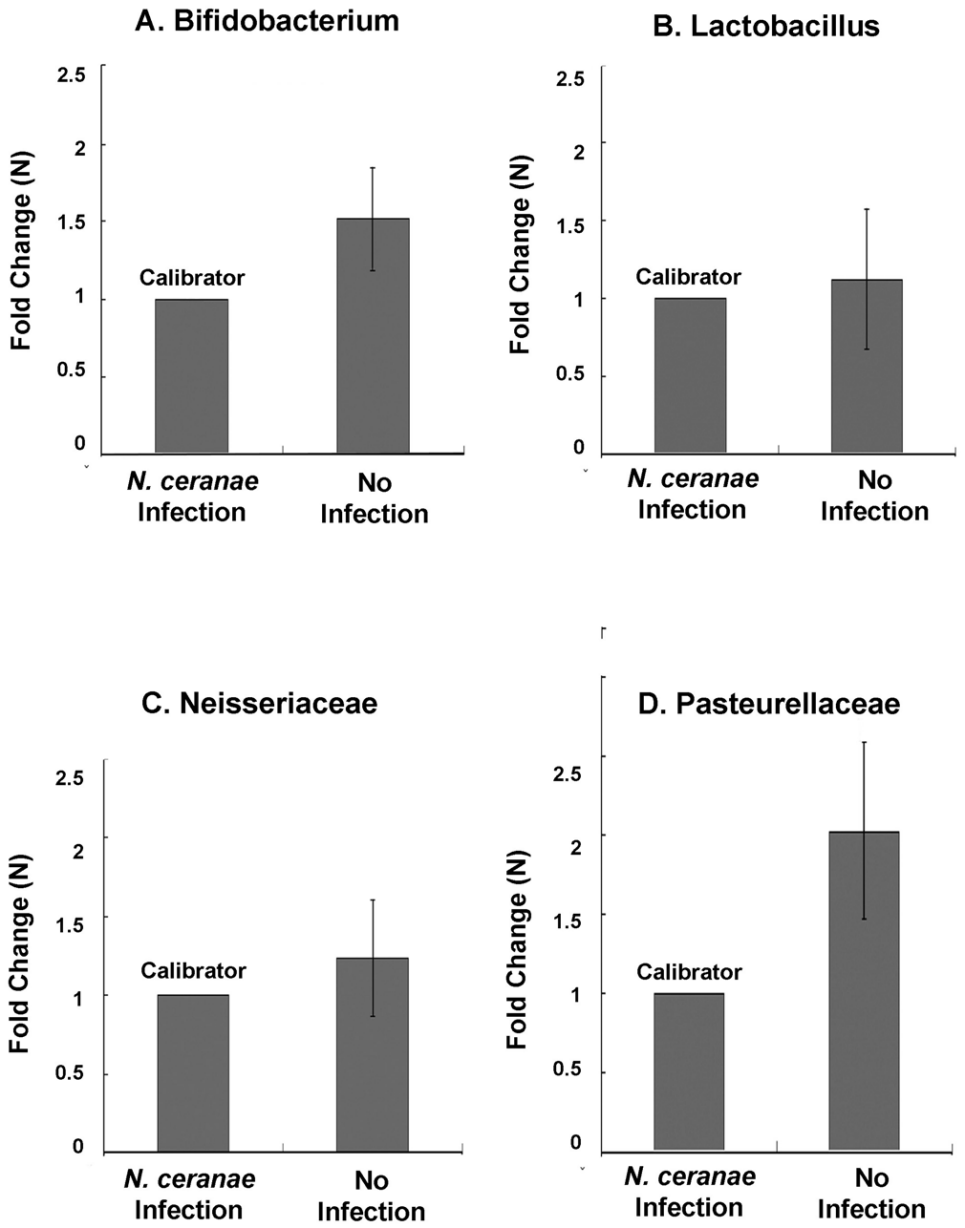
Relative abundance of four bacterial groups between *N. ceranae*-infected and non-infected *A. cerana*. A) Bifidobacterium; B) Lactobacillus, C) Neisseriaceae, and D) Pasteurellaceae. The y-axis depicts fold difference in quantities of each bacterial species between non-infected workers and *N. ceranae* infected workers. For each bacterial group, the *N. ceranae* infected workers had relatively lower level of bacterial titer, compared to non-infected bees and therefore was chosen as a calibrator. The concentration of each bacterial group in non-infected bees was compared with calibrator and expressed as n-fold change.

The quantities of these four bacterial taxa were compared between workers infected and non-infected by *N. ceranae*. The results showed that the quantities of *Bifidobacterium*, *Neisseriaceae* (t = 40.696, df = 2, p = 0.05), *Pasteurellaceae* and *Lactobacillus* (t = 10.362, df = 2, p = 0.09) in workers infected by *N. ceranae* tended to be lower, with infected workers having significantly lower *Bifidobacterium* (t = 51.924,df = 8,p = 0.0001) and *Pasteurellaceae* (t = 63.752, df = 8, p = 0.0001) quantities than non-infected workers (Figure 7A, B, C, and D).

## Discussion

We report the first survey of the prevalence of major honeybee pathogens in Asian honeybees *A. cerana* colonies from 19 provinces of China. The findings of this nationwide survey indicate that viral and parasitic pathogens including DWV, BQCV, *N. ceranae*, and *C. bombi* that have been implicated in population declines of European honeybees and bumble bees are widespread in populations of *A. cerana* and therefore are generalist pathogens of the genus *Apis*. However, the prevalence and infection profiles of pathogens and parasites differs significantly across different provinces in China, which likely reflects differences in plant species and climate conditions of different geographical locations.

The microsporidian intracellular parasite *Nosema* has been considered one of the most serious pathogens of domesticated honeybees and infection has negative impacts on worker longevity and colony health. Recent studies have suggested that *N. ceranae* is more common and virulent than *N. apis* at both the individual and colony levels of *A. mellifera*. In agreement with previous findings [14], [55]–[60], our survey further confirms that *N. ceranae* is a predominant species in Asian honeybees. *N. ceranae* was widespread in *A. cerana* colonies, with the highest prevalence being 98 percent. *N. apis* that was first described in *A. mellifera*, was not detected in any samples screened. The prevalence of *N. ceranae* infection differed among different regions of the country with Southern part of provinces had a relatively higher infection rate than Northern part of provinces, providing additional evidence that *N. ceranae* is less resistant in low temperature and its spread and virulence could be negatively influenced by cold climate [61].

This study provides the first evidence of *C. bombi* infection in *A. cerana*. Phylogenetic analysis showed that *C. bombi* isolated from bumble bees clustered together with the *C. bombi* isolates from northern populations of *A. cerana* while *C. bombi* from southern provinces form a clade that was distinct and as distant from the *C. bombi* clade as both were to the distant *A. mellifera* parasite *C. mellifera*. Future work will determine whether the isolates from the ‘*bombi*’ clade were recently acquired from bumble bee hosts. Interestingly, a negative correlation was found between the prevalence of *C. bombi* and that of *N. ceranae*. While this is only correlative, it could suggest parasite competition or trade-offs between host-defenses against the distinct parasites.

While the markers investigated were generally of conserved genomic regions, the phylogenetic analyses of *N. ceranae* and *C. bombi* revealed a level of diversity in the parasite populations of different geographic regions. Such diversity could have important implications for host-parasite dynamics, and further more in depth analyses may allow a greater understanding of the flow of these parasites between native and non-native pollinators.

DWV and BQCV appear to be the two most prevalent viral infections in *A. mellifera* [62]–[64]. Among the seven viruses we tested, only BQCV and DWV were ever detected in the *A. cerana* colonies. Of these two viruses, BQCV was the more prevalent virus in the *A. cerana* colonies. The incidence and prevalence of DWV infection in *A. cerana* appeared to be significantly lower, compared to *A. mellifera*. Previous work has shown transmission of DWV by *Varroa* mites [65] commonly associated with *A. mellifera* colonies. *A. cerana* has strong ability of resisting infestation of *Varroa* mites. Low levels of *Varroa* mite infestation could therefore explain the lower prevalence of DWV. The original host of the *Varroa* mite was the *A. cerana* from which spread to the poorly defended *A. mellifera*. Phylogenetic trees used to reconstruct evolutionary events showed that DWV isolated from *A. cerana* matched one of two distinct DWV lineages found in *A. mellifera*. Again, more work is needed to determine whether this result reflects a recent introduction of DWV from non-native *A. mellifera* or conversely historical movement from *A. cerana* into *A. mellifera*. An earlier study by Ai et al [25] found CBPV, IAPV and SBV in *A. cerana* colonies. The reason for the difference between this study and ours may be due to seasonal variation in the presence of viruses or due to the different sampling locations.

*Bifidobacterium*, *Neisseriaceae*, *Pasteurellaceae* and *Lactobacillus* were all present in the microbiota of the *A. cerana* samples examined. Interestingly, *N. ceranae* infected workers had different quantities of *Bifidobacterium* and *Pasteurellaceae*, with quantities being lower than in non-infected workers. As this is a correlative result, either *Nosema* infection could result in the reduction of these microbiota components or conversely the microbiota composition could determine the susceptibility to infection. Experimental studies, such as those already performed in bumblebees [44] will be required to work out the causal relationship. However, *Bifidobacterium* group produces several antibiotic compounds which could give this bacterium an interesting role in mediating parasite defense [45].

Our results give an overview of the distribution of parasites and pathogens of *A. cerana* across China. The patterns hint at a number of potentially interesting processes that could be going on, including between host-species transmission of parasites and a potentially key role of microbiota in mediating a host-parasite interaction. While this study lays a foundation, further investigation, including empirical work, is needed to further understand these processes that will have important implications for the health of the native honeybee *A. cerana* and the continued effectiveness of native bee species for apiculture in Asia.
